# Platelet-to-Hemoglobin Ratio Is an Important Predictor of In-Hospital Mortality in Patients With ST-Segment Elevation Myocardial Infarction

**DOI:** 10.7759/cureus.26833

**Published:** 2022-07-14

**Authors:** Ferhat Işık, Serdar Soner

**Affiliations:** 1 Department of Cardiology, University of Health Sciences, Gazi Yaşargil Training and Research Hospital, Diyarbakır, TUR

**Keywords:** myocardial infarction, in-hospital mortality, phr, hemoglobin count, platelet count

## Abstract

Background: Despite effective interventional treatments, the mortality of acute ST-segment elevation myocardial infarction (STEMI) is still high. Several mortality predictors are known in STEMI. Platelet-to-hemoglobin ratio (PHR) is a recently used mortality parameter in cardiac or non-cardiac diseases. We aim to investigate the relationship of PHR with in-hospital mortality in patients with STEMI.

Methods: Eight hundred eighty-four patients were included in the study. All of them underwent coronary intervention due to STEMI. Demographic characteristics, laboratory, electrocardiographic and echocardiographic parameters were analyzed from hospital records. A cut-off value for PHR was determined using receiver operating characteristic (ROC) curve analysis. Then, patients were divided into two groups PHR < 1.99 and PHR ≥ 1.99. The data of both groups were compared.

Results: The median age of the study population was 64 (54-75). Of these 633 (71.6 %) were male and 251 (28.4 %) were female. All cause mortality of the study population was 9.7% (n=86). In multivariable logistic regression analysis, PHR was independently associated with a significantly increased risk of in-hospital mortality for STEMI (OR: 2.645, CI: 1.641-4.263, p< 0.001). Also, age (OR: 1.044, CI: 1.021-1.067, p< 0.001), mean arterial pressure (MAP) less than 87 mmHg (OR: 2.078, CI: 1.185-3.645, p= 0.011), prior coronary artery disease (CAD) (OR: 2.839, CI: 1.345-5.993, p= 0.006), anterior myocardial infarction (MI) (OR: 1.912, CI: 1.069-3.421, p= 0.029), creatinine (OR: 3.710, CI: 2.255-6.106, p<0.001), alanine transaminase (ALT) (OR: 1.004, CI: 1.001-1.007, p=0.002), and neutrophil-to-lymphocyte ratio (NLR) (OR: 1.122, CI: 1.014-1.242, p= 0.025) were determined as independent predictors of in-hospital mortality.

Conclusion: In conclusion, we found that PHR is an independent predictor of in-hospital mortality in patients with STEMI.

## Introduction

Acute coronary syndromes (ACS) are the most common causes of death worldwide [1-3]. ST-segment elevation myocardial infarction (STEMI) is one of the leading types of ACS. Although STEMI mortality has decreased recently with effective primary percutaneous intervention, mortality remains high. The in-hospital mortality rate of STEMI patients in European registries ranges from 4% to 12%. It has been reported that the one-year mortality rate of STEMI patients is approximately 10% [4]. In a retrospective cross-sectional study in the United States, the 30-day mortality was 14.0%-14.6% [5]. Previous studies have shown that many parameters can predict in-hospital mortality in patients with STEMI. Recently, some hemogram parameters have been used to predict mortality and/or major cardiac events in studies on STEMI. Patients with STEMI and high platelet counts have poor in-hospital outcomes [6]. Increased mean platelet volume (MPV) in STEMI patients is associated with short-term mortality [7]. In addition, higher MPV-to-platelet count ratios were associated with long-term stent thrombosis and mortality in patients with STEMI [8]. Lower hemoglobin (< 13.1 g/dL) is associated with increased major adverse cardiovascular events (MACE) in STEMI [9]. Also, low baseline hemoglobin is a simple and powerful predictor of adverse outcomes in patients who underwent percutaneous coronary intervention (PCI) [10].

The significance of platelet-to-hemoglobin ratio (PHR) has been a matter of interest in recent years. PHR is a simple hemogram parameter that has been recently used to predict mortality in patients with coronary artery disease (CAD). Elevated PHR was associated with an increased risk of long-term all-cause mortality in patients with CAD and chronic heart failure (CHF) [11]. PHR is an independent predictor of adverse outcomes in patients with CAD who underwent PCI [12]. Although PHR is associated with adverse cardiac events in patients with CAD, its effect on in-hospital mortality in STEMI patients is unknown. In this study, we aim to investigate the relationship of PHR with in-hospital mortality in patients with STEMI.

## Materials and methods

**Figure 1:**
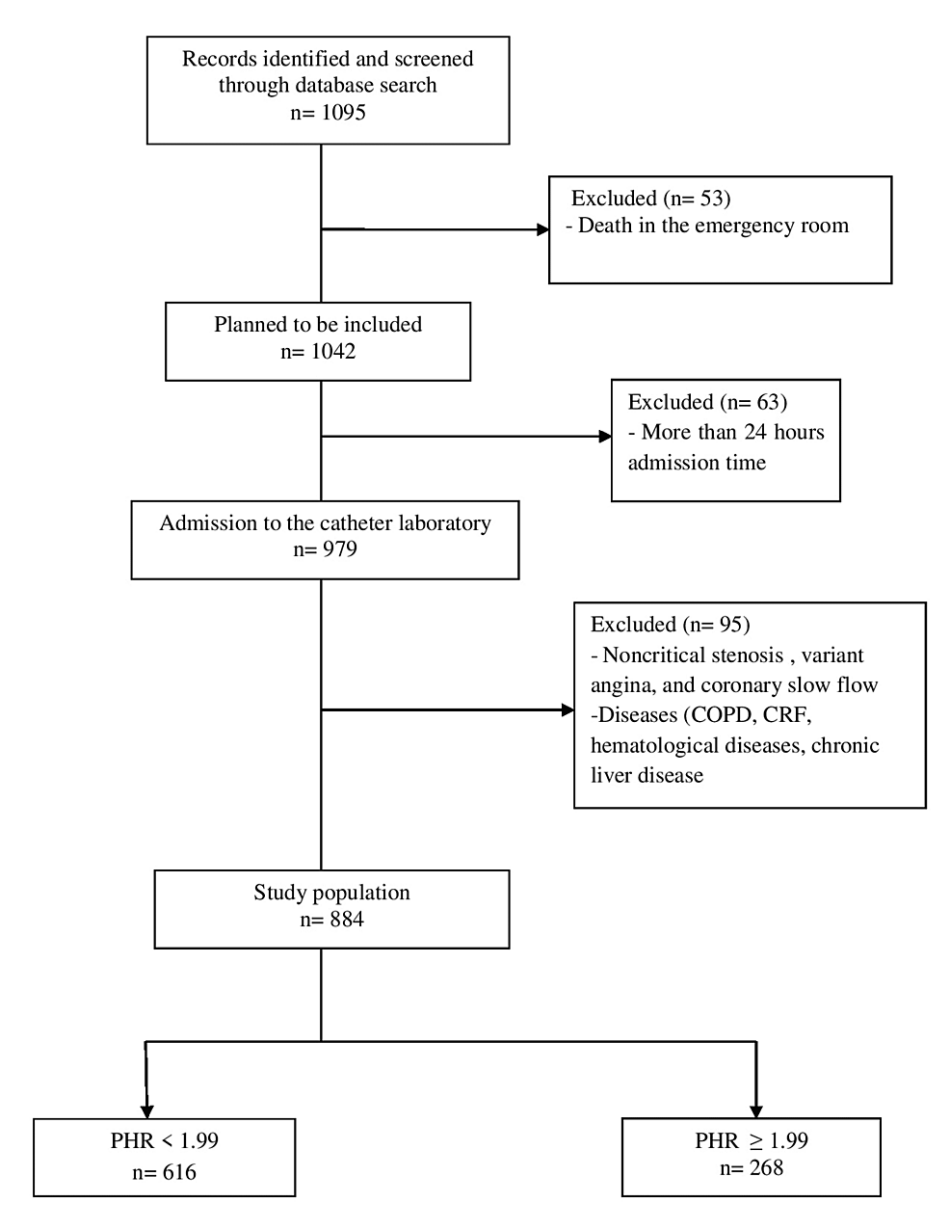
Flow-chart of patient selection. COPD: Chronic obstructive pulmonary disease, CRF: Chronic renal failure, PHR: Platelet-to-hemoglobin ratio

This is an observational, single-center, and retrospective study. The data that we used in this study was based on the electronic clinical management records system of the Diyarbakır Gazi Yaşargil Training and Research Hospital. The data of 1095 patients who applied to our emergency department with STEMI between June 1, 2018, and December 31, 2019, was scanned. Fifty-three of these patients died in the emergency room before being transferred to the catheter laboratory. Since the onset of symptoms in 63 patients were more than 24 hours, the acute intervention was not performed. Sixty patients had noncritical stenosis and/or coronary slow flow. We excluded thirty-five patients who had diseases such as hematological diseases, chronic liver disease, chronic obstructive pulmonary disease, and patients in need of renal replacement treatment (hemodialysis or peritoneal dialysis) that may affect platelet counts and hemoglobin levels. After exclusion criteria, data of the remaining 884 patients were collected (Figure 1). The baseline data for all patients included demographic characteristics, medical history, laboratory tests, electrocardiographic, and echocardiographic results. PHR was obtained by the ratio of platelet measured in 10^9^/L and hemoglobin measured in gram/L in the hemogram of admission [11]. The patients were receiving medical treatment according to current European Society of Cardiology guidelines [4]. PCI (stent implantation and/or balloon angioplasty) was performed in accordance with authoritative clinical practice guidelines [13]. The ethics committee approval required for our study was obtained from the ethics committee of our hospital (Protocol no: 94, date: 20.05.2022).

Statistical analysis

The histogram and Shapiro-Wilks test were used to confirm the normal or non-normal distribution of data. The continuous variables were presented as a median interquartile range (IQR) (25%-75%) owing to their non-normal distribution. The categorical variables were expressed as percentages. The chi-square test was used to compare categorical variables between groups. Continuous variables were compared by the Mann-Whitney U test. The cut-off value of the PHR for predicting in-hospital mortality was estimated by the receiver operating characteristic (ROC) curve analysis. The univariate and multivariate logistic regression analyses were performed to determine the predictors of in-hospital mortality. Variables with a p-value <0.05 were added to the model in multivariate analysis. Standardized β coefficients and 95% confidence intervals (CI) were calculated. The "p" value was used to interpret the statistical significance level of the received data. Statistical significance was defined as a value of p 0.05. The analysis of the data was carried out using IBM Corp. Released 2016. IBM SPSS Statistics for Windows, Version 24.0. Armonk, NY: IBM Corp.

## Results

**Figure 2:**
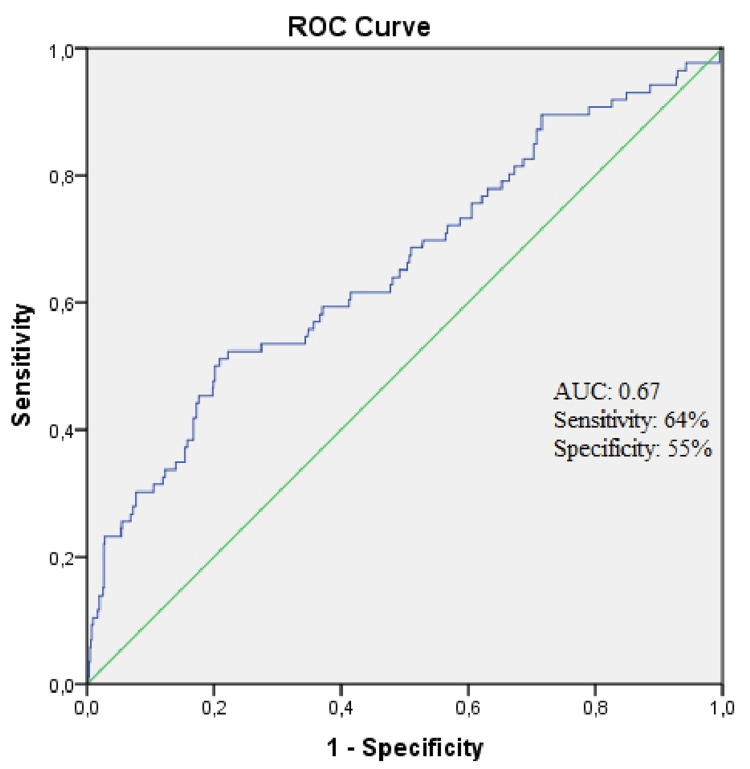
ROC curve for the platelet-to-hemoglobin ratio AUC: Area under the curve, ROC: Receiver operating characteristic

Eight hundred eighty-four patients were included in the study. The median age of the study population was 64 (54-75 IQR), and 633 (71.6 %) patients were male. The patients were divided into two groups Group 1 (PHR<1.99, n=616) and Group 2 (PHR ≥ 1.99, n=268). The ROC curve revealed 1.99 as the optimal cut-off value of PHR. Area under the curve (AUC): 0.67 (standard deviation: 0.033, 95% confidence interval; 0.59-0.72, sensitivity: 64%, specificity: 55%) (Figure 2). The total mortality of the study population was 9.7% (n=86). Age, mean platelet volume (MPV), white blood cell (WBC), potassium, and in-hospital mortality were higher in Group 2 compared to Group 1 (Respectively p= 0.035, p< 0.001, p= 0.002, p= 0.005, and p< 0.001). The male gender ratio was higher in Group 1 (p< 0.001). The study population's baseline characteristics and clinical and laboratory findings are presented comparatively in Table 1. About half of the deaths were due to fatal arrhythmias and reinfarctions (n=41). Twenty-three patients died from major bleeding (cerebrovascular hemorrhage, gastrointestinal bleeding, etc.). Twenty-two patients died from other causes (such as ventricular pump failure, shock, chordae tendinea rupture, papillary muscle rupture, ventricular septal defect, ischemic stroke, acute renal failure, and infection).

The independent predictors of mortality were determined by univariable and multivariable logistic regression analysis. In multivariable analysis, PHR was independently associated with a significantly increased risk of in-hospital mortality of STEMI (OR: 2.645, CI: 1.641-4.263, p< 0.001). Also, age (OR: 1.044, CI: 1.021-1.067, p< 0.001), mean arterial pressure (MAP) less than 87 mmHg (OR: 2.078, CI: 1.185-3.645, p= 0.011), prior coronary artery disease (CAD) (OR: 2.839, CI: 1.345-5.993, p= 0.006), anterior myocardial infarction (MI) (OR: 1.912, CI: 1.069-3.421, p= 0.029), creatinine (OR: 3.710, CI: 2.255-6.106, p<0.001), alanine transaminase (ALT) (OR: 1.004, CI: 1.001-1.007, p=0.002), and neutrophil-to-lymphocyte ratio (NLR) (OR: 1.122, CI: 1.014-1.242, p= 0.025) were determined as independent predictors of in-hospital mortality (Table 2). 

**Table 1:** Baseline characteristics and laboratory findings of the study population Data are expressed as median, interquartile range and count (percentage). ALT: Alanine transaminase, AST: Aspartate transaminase, CAD: Coronary artery disease, CHF: Chronic heart failure, CRF: Chronic renal failure, HDL: High-density lipoprotein, LDL: Low-density lipoprotein, LVEF: Left ventricle ejection fraction, MI: Myocardial infarction, MPV: Mean platelet volume, NLR: Neutrophil-to-lymphocyte ratio, PHR: Platelet-to-hemoglobin ratio, WBC: White blood cell

Variables	Total (n=884)	Group 1 (PHR <1.99)(n= 616)	Group 2 (PHR >1.99)(n=268)	p value
Age, years	64(54-75)	63(53-73)	65(55-76)	0.035
Gender, male, n (%)	633(71.6)	475(77.1)	158(58.9)	<0.001
Prior CAD, n (%)	97(10.9)	65(10.5)	32(11.9)	0.559
Prior CHF, n (%)	57(6.5)	39(6.3)	18(6.7)	0.882
Diabetes mellitus, n (%)	264(29.8)	177(28.7)	87(32.4)	0.264
Hypertension, n (%)	309(34.9)	205(33.3)	104(38.8)	0.125
MI type				
Anterior, n (%)	426(48.2)	291(47.2)	135(50.3)	0.421
Other, n (%)	458(51.8)	325(52.8)	133(49.7)	0.436
Heart rate, beat/min	84(76-92)	84(76-92)	84(75-91)	0.481
Mean arteriel pressure, mmHg	86.6(80.0-93.0)	86.6(80.0-93.0)	86.6(80.0-93.0)	0.938
LVEF, %	50(40-55)	50(40-55)	50(40-55)	0.798
Glucose, mg/dL	135(106-184)	134(105-183)	140(110-185)	0.191
Creatinine, mg/dL	0.86(0.79-1.05)	0.84(0.79-1.01)	0.88(0.79-1.06)	0.635
Sodium, meq/L	139(137-140)	139(137-140)	139(137-140)	0.311
Potassium, meq/L	4.17(3.80-4.52)	4.10(3.77-4.50)	4.20(3.80-4.80)	0.005
AST, IU/L	21(15-33)	22(16-33)	21(14-33)	0.296
ALT, IU/L	29(20-54)	29(20-54)	28(20-54)	0.373
Total cholesterol, mg/dL	178(155-204)	176(153-204)	183(157-206)	0.212
HDL, mg/dL	34(30-39)	34(29-40)	34(30-39)	0.813
LDL, mg/dL	118(95-136)	113(95-135)	122(99-137)	0.179
Triglyceride, mg/dL	114(78-159)	115(78-158)	110(82-174)	0.541
Troponin, ng/ml	1.72(0.35-13.72)	1.57(0.33-11.27)	2.43(0.45-15.01)	0.105
WBC, 10^9^/ L	11.63(9.58-13.74)	11.39(9.38-13.44)	11.98(9.85-14.96)	0.002
NLR	3.60(2.16-6.40)	3.64(2.11-6.40)	3.56(2.40-6.17)	0.981
MPV, fL	10.1(9.50-10.8)	9.8(8.9-10.3)	10.3(9.7-10.9)	<0.001
In-hospital exitus, n (%)	86(9.7)	40(6.5)	46(17.1)	<0.001

**Table 2:** Mortality predictors with univariate and multivariate regression models. ALT: Alanine transaminase, AST: Aspartate transaminase, CAD: Coronary artery disease, CHF: Chronic heart failure, DM: Diabetes mellitus, EF: Ejection fraction, MAP: Mean arterial pressure, MI: Myocardial infarction, NLR: Neutrophil-to-lymphocyte ratio, PHR: Platelet-to-hemoglobin ratio, PLR: Platelet-to-lymphocyte ratio, WBC: White blood cell.

	Univariate logistic analysis			Multivariate logistic analysis		
Covariates	Odds ratio	95% Confidence Interval	p value	Odds ratio	95% Confidence Interval	p value
Age, years	1.048	1.030-1.066	<0.001	1.044	1.021-1.067	<0.001
MAP < 87 mmhg	1.581	1.012-2.471	0.004	2.078	1.185-3.645	0.011
Prior CAD	2.211	1.240-3.945	0.007	2.839	1.345-5.993	0.006
Anterior MI	2.042	1.287-3.240	0.002	1.912	1.069-3.421	0.029
Female sex	1.952	1.237-3.081	0.004	1.345	0.731-2.474	0.341
Prior CHF	3.052	1.571-5.926	0.001	1.042	0.366-2.963	0.939
DM	2.228	1.419-3.498	<0.001	1.127	0.532-2.386	0.755
EF	0.950	0.928-0.971	<0.001	0.969	0.935-1.004	0.080
Glucose	1.004	1.002-1.006	<0.001	1.003	1.000-1.007	0.051
Creatinine	4.569	3.022-6.908	<0.001	3.710	2.255-6.106	<0.001
Potassium	1.907	1.375-2.644	0.001	0.913	0.589-1.415	0.683
AST	1.005	1.002-1.007	0.001	0.996	0.991-1.000	0.076
ALT	1.003	1.002-1.005	0.001	1.004	1.001-1.007	0.002
WBC	1.145	1.084-1.209	<0.001	0.946	0.856-1.046	0.281
PLR	1.004	1.002-1.006	0.001	0.995	0.990-1.001	0.094
NLR	1.091	1.054-1.129	<0.001	1.122	1.014-1.242	0.025
PHR	2.374	1.758-3.205	<0.001	2.645	1.641-4.263	<0.001

## Discussion

The significance of biomarkers on prognosis in cardiovascular diseases has been a matter of interest in recent years. To the best of our knowledge, this is the first study to investigate the relationship between in-hospital mortality and PHR in patients with STEMI. Our study found that increased PHR was an independent predictor of in-hospital mortality in patients with STEMI.

Despite the development of mechanical revascularization treatments, especially in infarct-related arteries, STEMI is still the main cause of morbidity and mortality worldwide [14]. The relationship between some clinical predictors and in-hospital mortality have shown in many studies in patients with STEMI. The most known of these predictors are; elderly (age ≥65 years), acute heart failure (Killip class III-IV), heart rate, systolic blood pressure, total myocardial ischemia time ≥3 hours, anterior MI, failure of PCI, cardiac arrest, peripheral arterial disease, prior MI, prior CHF, SYNTAX scale score ≥16, elevated initial serum creatinine levels, glycemia on admission ≥7.78 mmol/l for patients without a history of diabetes mellitus (DM) and ≥14.35 mmol/l for patients with a history of DM [15,16,17]. The prognostic value of various hemogram parameters in predicting adverse outcomes of cardiovascular diseases is also well known [18]. Avcı et al. reported that among hemogram parameters (such as WBC, red cell distribution width, MPV, and NLR), WBC and especially NLR were more associated with increased risks of in-hospital mortality in patients with STEMI [19]. Additionally, it is known that NLR and WBC may be independent factors in determining the mortality of STEMI [20-22]. In our study, parameters such as age, anterior MI, NLR, creatinine, low blood pressure, and prior CAD were consistent with in-hospital mortality predictors determined in previous “mortality in STEMI” studies.

High platelet counts are associated with adverse outcomes in cardiovascular disease [23]. It has been reported that the pathogenesis of this may be inflammatory response and platelet activation [24]. Low hemoglobin level is associated with adverse cardiovascular events [25]. PHR was shown as a novel prognostic predictor for cardiovascular disease. Zheng et al. found that PHR was an independent prognostic marker for CAD patients after PCI with better prognostic value than absolute platelet counts or hemoglobin levels. PHR higher than 1.92 is associated with all-cause mortality, cardiac mortality, re-admission, and MACE and/or cerebrovascular events in these patients [12]. Bao et al. showed that a PHR higher than 1.69 was correlated with an increased risk of long-term all-cause mortality in patients with CAD and CHF [11]. In our study, we found that in-hospital mortality increased more than 2.5 times in patients with a PHR above 1.99.

The mechanism of increased PHR in STEMI patients is not clear. There are some potential mechanisms for this topic. First, inflammation and thrombus formation are known in the pathogenesis of ACS. Unstable atherosclerotic plaque rupture seen after endothelial injury in STEMI creates a prothrombotic condition. During a proinflammatory condition such as acute MI, a large number of mediators are released, resulting in megakaryocyte proliferation and an increase in platelet count in the blood. This could imply a prothrombotic situation and significant platelet activity [26]. Interleukin‑6 (IL‑6), IL‑8, and CD40 ligands were found to regulate monocyte tissue factor production. Thus, activation of the extrinsic coagulation cascade occurs [19]. In another mechanism, low hemoglobin concentration in circulation indicates decreased oxygen-carrying capacity, which may impair myocardial ischemic injury. Anemia can raise complete blood aggregometry, which can artificially promote platelet aggregation. Hemoglobin deficiency is frequently linked to inflammation. Inflammatory indicators, including fibrinogen, von Willebrand factor, and inflammatory cytokines, may directly increase platelet reactivity. Also, due to the anemic environment, the bone marrow turns into hyperactivity and then secretes more platelets [27]. We can say that there is a potential relationship between high platelet count and low hemoglobin. The results of our study were consistent with platelet, hemoglobin, and PHR results in other CAD studies. Considering all these, our study supports that increased PHR is associated with in-hospital mortality in patients with STEMI.

Limitations

There are some limitations of our study. The first and most important one, this study was a single retrospective cohort design. Therefore, our results must be further verified in a multicenter, prospective study. Platelet counts and hemoglobin levels at admission were included in our study. Hence, we did not know the changes in platelet count and hemoglobin levels during hospitalization. This may have affected in-hospital mortality. Also, we could not reach the information of all patients about smoking and Killip classifications. Therefore, we did not include smoking and Killip scores in our study. Although some studies in the literature that a high Killip score adversely affects mortality, we did not know how it impacted our study. Finally, the gender differences were significant between groups. Gender differences were also an important limitation parameter in this study.

## Conclusions

High PHR is associated with an increased risk of in-hospital mortality in STEMI. In conclusion, these results indicate that PHR may be a useful prognostic biomarker for STEMI patients. It can be beneficial for clinicians to follow closely platelet counts and hemoglobin levels in this population.
